# Endocrine-Mediated Mechanisms of Metabolic Disruption and New Approaches to Examine the Public Health Threat

**DOI:** 10.3389/fendo.2019.00039

**Published:** 2019-02-07

**Authors:** Christopher D. Kassotis, Heather M. Stapleton

**Affiliations:** Nicholas School of the Environment, Duke University, Durham, NC, United States

**Keywords:** endocrine disrupting chemicals, obesogen, diabetogen, adipogenesis, 3T3-L1, obesity, diabetes

## Abstract

Obesity and metabolic disorders are of great societal concern and generate substantial human health care costs globally. Interventions have resulted in only minimal impacts on disrupting this worsening health trend, increasing attention on putative environmental contributors. Exposure to numerous environmental contaminants have, over decades, been demonstrated to result in increased metabolic dysfunction and/or weight gain in cell and animal models, and in some cases, even in humans. There are numerous mechanisms through which environmental contaminants may contribute to metabolic dysfunction, though certain mechanisms, such as activation of the peroxisome proliferator activated receptor gamma or the retinoid x receptor, have received considerably more attention than less-studied mechanisms such as antagonism of the thyroid receptor, androgen receptor, or mitochondrial toxicity. As such, research on putative metabolic disruptors is growing rapidly, as is our understanding of molecular mechanisms underlying these effects. Concurrent with these advances, new research has evaluated current models of adipogenesis, and new models have been proposed. Only in the last several years have studies really begun to address complex mixtures of contaminants and how these mixtures may disrupt metabolic health in environmentally relevant exposure scenarios. Several studies have begun to assess environmental mixtures from various environments and study the mechanisms underlying their putative metabolic dysfunction; these studies hold real promise in highlighting crucial mechanisms driving observed organismal effects. In addition, high-throughput toxicity databases (ToxCast, etc.) may provide future benefits in prioritizing chemicals for *in vivo* testing, particularly once the causative molecular mechanisms promoting dysfunction are better understood and expert critiques are used to hone the databases. In this review, we will review the available literature linking metabolic disruption to endocrine-mediated molecular mechanisms, discuss the novel application of environmental mixtures and implications for *in vivo* metabolic health, and discuss the putative utility of applying high-throughput toxicity databases to answering complex organismal health outcome questions.

## Endocrine Disruptors as Causative Factor in Metabolic Disruption

**Figure 1:**
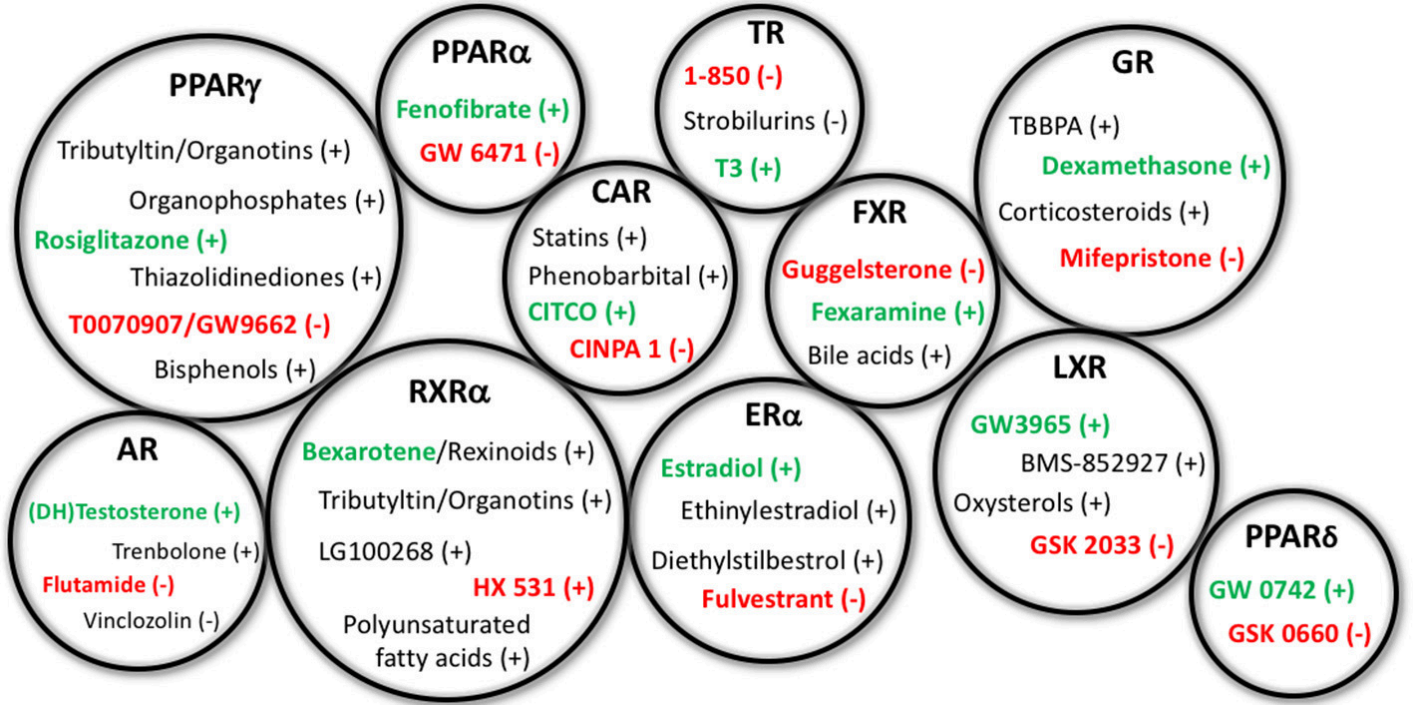
Representative EDCs Capable of Affecting Adipogenesis. Representative endocrine disrupting chemicals (EDCs) capable of affecting adipogenesis and/or metabolic health through the specified nuclear receptor pathways listed above. Gross circle size intended to express a general sense of the reported research into assessing these varying mechanisms; for example, PPARγ, RXRα, and GR have previously received the bulk of the research, whereas others have received less. Agonists for the receptors are depicted with a (+) following the chemicals, whereas antagonists are denoted with the (−). Standard positive and negative control chemicals for each receptor (for evaluating these pathways) are bolded to distinguish from the other EDC examples. PPAR, peroxisome proliferator activated receptor; RXR, retinoid X receptor; AR, androgen receptor; ER, estrogen receptor; CAR, constitutive androstane receptor; TR, thyroid receptor; FXR, farnesoid X receptor; LXR, liver X receptor; GR, glucocorticoid receptor.

Endocrine disrupting chemicals (EDCs) have been demonstrated to directly modulate metabolism *in vivo* and/or triglyceride accumulation *in vitro* through various receptor-mediated pathways (1–5), suggesting a potential causative link between exposure to EDCs and the increasing global prevalence of metabolic disorders, including obesity (6). Chronic metabolic health conditions are rapidly increasing in prevalence and cost to society worldwide: in the US, 39.6 and 9.7% of adults aged 20 and older are currently classified as obese or have been diagnosed with diabetes, respectively, with increasing occurrence in younger age groups as well (7–10). These conditions contribute to a rising share of health care costs; in the US, >$600 million is directed to obesity-related and diabetes-related illnesses in adults (10, 11). These effects are mirrored in animal populations, with an analysis of >20,000 animals from 24 populations reporting increased weight gain in numerous species including monkeys, both laboratory and urban mice, cats, dogs, etc. (12). Notably, attempted interventions have yielded minimal effects, and analyses have determined that activity, caloric intake, and genetics are insufficient to explain the magnitude and speed of this change (13, 14). As fat cell development is driven and modulated by nuclear hormone receptor signaling (2, 15–17), EDCs that activate or inhibit these hormone pathways may be causative agents in promoting modulation of fat cell development, energy homeostasis, basal metabolic rate, hormonal control of appetite and satiety, and brain circuitry controlling food intake and energy expenditure and ultimately contributing to the development of Metabolic Syndrome (Figure 1) (18).

Numerous environmental toxicants have been demonstrated as metabolic disruptors *in vivo*, supporting EDCs as a causative factor in these adverse health trends (14). There is a rich literature demonstrating effects of antibiotics on weight gain in humans and diverse animal species. Experiments demonstrating their efficacy in promoting weight gain in agricultural species were published by the 1950's, presumably operating through effects on gut microbiota impacting the processing of carbohydrates in the diet (19, 20). More recent publications have demonstrated that several weeks of subtherapeutic antibiotics increase fat mass and weight, particularly when begun during gestation (21, 22), and human epidemiological studies have demonstrated increased risk of becoming overweight when children were exposed early in life (23, 24). Other notable examples include diethylstilbestrol (DES), a pharmaceutical provided to pregnant women in the 1940's through 1970's in the mistaken assumption it would reduce rates of abortion, miscarriage, and premature labor (25); it was later determined to induce a variety of adverse health effects in both males and females exposed during gestation (26–29). DES has been demonstrated to promote triglyceride accumulation *in vitro*, seemingly through an estrogen-receptor mediated mechanism (30), and both gestational and perinatal DES exposure increases body weight, body fat, and alters serum lipid profiles in rodent models throughout life (31–33). Increased risks of obesity in human adults exposed prenatally to DES have also been reported (34), delineating apparent translational effects. Our lab has recently demonstrated that common chemicals and environmental mixtures associated with unconventional oil and gas (UOG) operations can activate the peroxisome proliferator activated receptor gamma (PPARγ) and promote triglyceride accumulation and pre-adipocyte proliferation *in vitro* (35), and that gestational exposure to a mixture of UOG chemicals resulted in increased body weights through weaning in a rodent model (36, 37). UOG development has also been associated with increased prevalence of low birth weight and small for gestational age births in the Northeast US (38), and decreased prevalence of low birth weights and increased risk of higher birth weight babies in Colorado (39); both low (40, 41) and high (42, 43) birth weights are associated with greater risks for obesity later in life.

As costs associated with *in vivo* screening of putative metabolism disruptors are prohibitively high, utilizing lower-order testing, and screening is essential to narrow higher-order testing to chemicals most likely to be active. Various pre-adipocyte and mesenchymal stem cell models (both rodent and human, primarily) have been utilized to assess putative *in vivo* metabolic disruptors *in vitro*; 3T3-L1 mouse pre-adipocytes have proven reliable as an *in vitro* screen for identifying likely obesogenic chemicals *in vivo*, and other models such as the OP9 mouse bone marrow-derived stromal pre-adipocyte cell line (44, 45) allow for assessments of varying molecular pathways important for the process of differentiation. Additionally, various multipotent mesenchymal cells and cell lines (46, 47) offer the additional ability to assess commitment to the adipocyte lineage as a distinct process from adipocyte differentiation (48). However, these assays are lengthy and their relative abilities to correctly identify chemicals may depend on both cell line and cell source. As such, there is a critical need to develop better methods for correctly predicting metabolic disruptors. Several high-throughput (HTP) screening programs now exist (Tox21, ToxCast) that report activity across mechanisms known to modulate metabolic health for thousands of chemicals. Harnessing these data sets to broadly assess high-scoring chemicals (across these molecular pathways) for more targeted *in vitro* and *in vivo* testing could provide a valuable tool for reducing research costs and more broadly assessing the tens of thousands of commercial chemicals for potential contributions to adverse health outcomes in humans and/or animals.

In addition to high-throughput screening, assessments of mixtures have become more commonplace in recent years. Tools to evaluate the chemical constituents and biological activities associated with complex environmental mixtures have vastly increased the capabilities within this sphere, though standard approaches to mixtures are still lacking in many respects, particularly in terms of relevance to human and animal exposure. One notable mixture that has received increasing attention is indoor house dust; our laboratory and others have collected and analytically characterized house dust from different environments around the world and routinely report numerous classes of EDCs (known to be hormonally active), including flame retardants, phthalates, pesticides, perfluoroalkyl substances (PFAS), and others that span a wide range of concentrations (49–51). Humans, and perhaps most importantly small children, are chronically exposed to household dust, and thus receive exposure to EDCs present in the dust. The EPA estimates children ingest 60–100 mg of dust per day from indoor environments (52), contributing to chronic oral and inhalation exposures to EDCs (49, 53, 54), and compounded by other routes of exposure. Notably, numerous studies have demonstrated clear links between levels of indoor semi-volatile indoor contaminants (SVOCs) on hand wipes with levels in house dust (55, 56), with other studies demonstrating clear links with urinary and serum levels (55, 57, 58), providing evidence for this exposure route contributing to an increased body burden of specific chemicals. As such, environmental matrices such as this may represent a clear exposure route for humans and could provide critical information on biological effects of summed mixture exposures.

## Nuclear Receptor Mechanisms Mediating Metabolic Disruption

**Figure 2:**
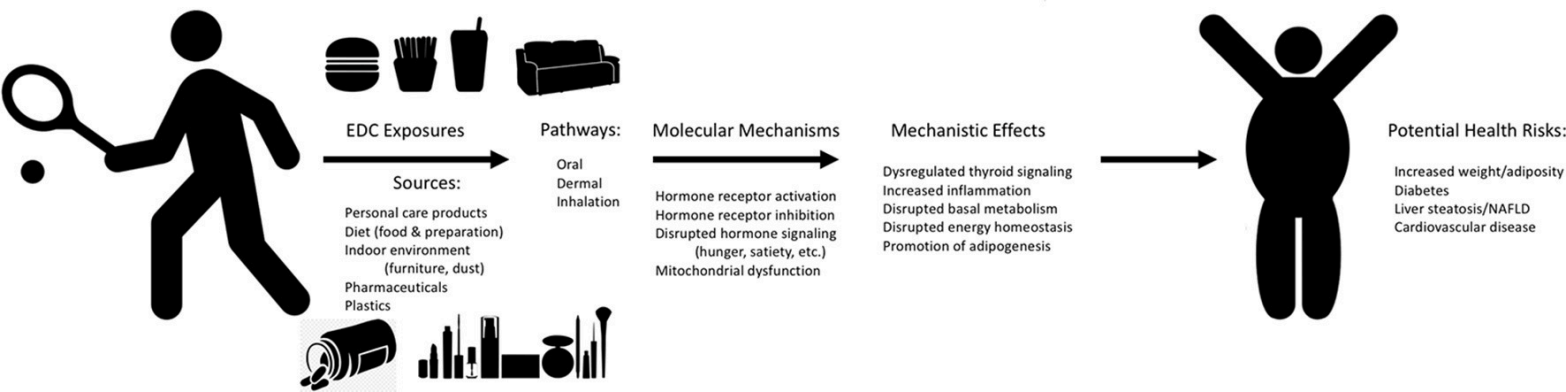
Mechanisms of EDC Exposure and Potential Human Metabolic Health Effects. Graphical depiction of the potential sources and exposure pathways for humans to endocrine disrupting chemicals (EDCs), the molecular mechanisms related to metabolic health through which these EDCs may act to drive specific mechanistic effects, all of which may contribute to potential adverse health risks for humans. Effects reported are representative and are not comprehensive to all molecular mechanisms and mechanistic effects.

While activation of the peroxisome proliferator activated receptor gamma (PPARγ) is likely the best-described mechanism through which adipogenesis is initiated/promoted, activation or inhibition of numerous other receptor systems have been described to directly or indirectly modulate adipocyte lineage commitment and/or differentiation of pre-adipocytes and subsequent accumulation of triglycerides, including thyroid receptor-beta (TRβ), glucocorticoid receptor (GR), estrogen receptor (ER), androgen receptor (AR), liver X receptor (LXR), retinoid X receptor (RXR), and others (59) (Table 1). Several studies have assessed the expression of nuclear receptors throughout the differentiation process, reporting that 30 nuclear receptors were expressed throughout the differentiation process to varying degrees and at varying timepoints (15, 60). Recent work by Chappell et al. demonstrated putative GR-mediated effects prior to PPARγ activation after exposure of 3T3-L1 cells to tetrabrominated bisphenol A (TBBPA) (61). Notably, EDCs capable of acting through each of these pathways have been described previously to modulate metabolic health *in vitro, in vivo*, or in human epidemiological studies; though importantly, certain molecular mechanisms have received far greater research attention than others (Figure 2).

**Table 1:** Major hormone receptor pathways capable of promoting adipogenesis.

**Receptor**	**Activity**	***In vitro* effects**	***In vivo* effects**	**Epidemiological effects**
PPARγ	Agonism	Promotes adipocyte differentiation, also some promotion of pre-adipocyte proliferation	Increased adipose fat deposition, body weights	Increased body weights, reverse hyperglycemia/treat diabetes
PPARβ/δ	Agonism	Promotes adipocyte differentiation	Activation improves lipid profiles, depletes lipid accumulation, increases resistance to diet-induced obesity	PPARβ/δ agonists reduce LDL cholesterol, triglycerides, insulin, and increase HDL cholesterol^*^
PPARα	Agonism	Promotes adipocyte differentiation	Activation improves hyperinsulinemia and hyperglycemia, reduces weight and adiposity	PPARα agonists reduce serum triglycerides and LDL cholesterol, increase HDL cholesterol
RXRα	Agonism	Promotes adipocyte lineage commitment, adipocyte differentiation	Ablated RXR mice are resistant to diet/chemical-induced obesity	RXR agonists increase plasma triglycerides, cholesterol, decreased thyroid hormones
GR	Agonism	Promotes adipocyte differentiation, pre-adipocyte proliferation	GR knock-down mice are resistant to diet-induced obesity, have improved insulin sensitivity and glucose tolerance, and increased energy expenditure	Excess glucocorticoids associated with increased weight, adiposity, and decreased glucose tolerance/insulin sensitivity
TR	Antagonism	Promotes adipocyte differentiation	TR null mice exhibit increased adipogenesis	Low thyroid hormone levels promote weight gain, high levels promote weight loss
ER	Agonism	Inhibits adipocyte differentiation, promotes pre-adipocyte proliferation	ERKO mice exhibit increased adiposity	Decreased estrogen in menopause associated with increased abdominal obesity
AR	Antagonism	Promotes adipocyte differentiation, no effect on pre-adipocyte proliferation	AR agonism has anti-adipogenic effects in rodents	Low androgen levels associated with increased abdominal obesity, reversed with supplementation
LXR	Agonism	Promotes adipocyte differentiation, pre-adipocyte proliferation	LXR knockout mice exhibit less adipose and are glucose-intolerant; agonist treatment reduces energy expenditure	LXR agonist treatments increase triglycerides, cholesterol, and other negative molecular markers
PXR	Agonism	Promotes adipocyte differentiation	PXR ablation inhibits diet-induced obesity, insulin resistance, and fatty liver disease; agonist treatment promotes adiposity in mice	PXR agonist treatments reported to induce hyperglycemia and increase diabetes risk
CAR	Agonism	Promotes adipocyte differentiation	CAR agonist treatment enhances insulin sensitivity, improves glucose and lipid metabolism, reverses diet-induced obesity	CAR agonist treatment decreases plasma glucose and improves insulin sensitivity
FXR	Agonism	Agonists induce adipocyte differentiation, antagonists reverse	FXR agonist induces weight gain and glucose intolerance in mice	FXR agonist treatments promote reduced lipid accumulation and increased glucose uptake, reduced HDL and increased LDL cholesterol, improved insulin sensitivity
InsR	Agonism	Promotes adipocyte differentiation, triglyceride accumulation	Increased weight gain and glucose intolerance	Insulin supplementation promote increased weight gain, cholesterol, and blood pressure
IGFR	Agonism	Promotes adipocyte differentiation, triglyceride accumulation	Increased weight gain and glucose intolerance	Increased weight gain, triglycerides

*Descriptive effects for several major hormone receptor pathways that influence the process of adipogenesis and weight maintenance. Summarized evidence is provided for direction of effects, as well as in vitro, in vivo, and human epidemiological evidence. References and more detailed descriptions can be found within the relevant subsections of the manuscript, within section Nuclear Receptor Mechanisms Mediating Metabolic Disruption*.

* *Due to lack of specific, potent, and available ligands, there is minimal reported work in humans. Summarized work describes effects observed in monkey models following treatment with receptor-specific agonists*.

### Peroxisome Proliferator Activated Receptors (PPARs)

PPARγ is often considered the only nuclear receptor whose activation is necessary and sufficient to initiate adipogenesis (62, 63). Treatment of 3T3-L1 cells as well as other pre-adipocyte and/or other committed adipocyte lineage cells with PPARγ agonists induces a potent and efficacious increase in triglyceride accumulation, which has long been realized (64, 65); as such, PPARγ agonists such as rosiglitazone and/or troglitazone are routinely utilized as positive control ligands for these assays (63). Utilized as therapeutic agents to treat type 2 diabetes, these thiazolidinediones may act to improve insulin sensitivity via induction of PPARγ in diverse tissue types, proliferation of smaller adipocytes that are more insulin-sensitive, or via mediation of the tumor necrosis factor alpha (TNF-α), leptin, or fatty acid signaling pathways [reviewed in (66)]. To establish the necessity of this pathway to adipogenesis, Rosen et al. utilized embryonic stem cell and chimeric mouse models. They demonstrated that PPARγ-null cells tended to not generate adipocytes, suggesting an essential role for this receptor in their formation. They further demonstrated gene dosage effects *in vitro*; cells lacking both copies of PPARγ could not be induced to differentiate, cells with one copy exhibited an intermediate degree of differentiation, and wild-type cells exhibited robust differentiation and efficacious expression of adipocyte-specific molecular markers (62). Clonal expansion and growth arrest occurs concurrently with expression of two proteins, PPARγ and the CCAAT enhancer binding protein alpha (C/EBPα), and these markers are both important for the differentiation of pre-adipocytes to adipocytes (63). Further experiments in this laboratory demonstrated that while C/EBPα is also a primary marker for the initiation of differentiation, it operates within a single initiating pathway with PPARγ (67). In cells deficient of PPARγ, C/EBPα was not capable of promoting adipogenesis by itself, suggesting an important but non-essential role in inducing and maintaining PPARγ expression, as well as an accessory role in mediating insulin sensitivity via direct induction of the insulin receptor (67).

The other PPAR isoforms, α and β/δ, have received considerably less attention as it relates to adipogenesis, though gain/loss-of-function experiments suggest putative roles. Experiments *in vitro* demonstrated that induction with a PPARβ/δ-specific ligand induced robust triglyceride accumulation in wild type cells (68) [and *in vivo* (69)], while PPARβ/δ-null cells differentiate and accumulate triglycerides less efficaciously following PPARγ-mediated induction (68). This suggests that while PPARβ/δ is not necessary for adipogenesis, the interplay of these isoforms is necessary to induce maximal differentiation and triglyceride accumulation in adipocytes. This was supported by other work reporting that PPARβ/δ activation promotes PPARγ expression, potentially bolstering adipogenesis, and providing a supportive role (70, 71). When examining the isoforms in isolation, Brun et al. reported that receptor isoform-specific activation via ligands failed to induce adipogenesis and triglyceride accumulation for PPARβ/δ, but did for PPARα, if to a lesser extent and over a longer time-course than via activation of PPARγ (72). RNA isolations on day five demonstrated that PPARα-treated cells had minimal or no induction of various adipocyte markers, relative to robust induction in the PPARγ-treated cells; however, both had robust expression by day seven post-induction, while PPARβ/δ exhibited only minimal expression at much later time points (72). Further experiments demonstrated that C/EBPα acted cooperatively with PPARγ to stimulate adipogenesis as expected, but not with PPARα or β/δ (72), suggesting distinct mechanisms. PPARβ/δ activation in mice has anti-adipogenic effects, improving lipid profiles, reducing lipid accumulation, and increasing resistance to diet-induced obesity (73, 74). PPARα activation in mice has similarly been demonstrated to result in anti-adipogenic effects, including improved hyperinsulinemia and hyperglycemia, lowered triglycerides, increased resistance to diet-induced obesity, and decreased weight and adiposity (75–77). Fenofibrates (PPARα agonists) administered to humans have similarly been demonstrated to decrease serum triglycerides and LDL cholesterol and increase HDL cholesterol (78, 79). While absence of good selective PPARβ/δ agonists has hindered human therapeutic examination, limited work with a selective and potent PPARβ/δ agonist in rhesus monkeys reported lowered LDL cholesterol, triglycerides, insulin, and increased HDL cholesterol (80).

### Retinoid X Receptor (RXRα)

PPARγ functions as a heterodimer with RXR, suggesting that this receptor might also have dependent and/or independent roles in adipogenesis (62). Indeed, more than a decade ago, it was reported that organotins, potent activators of both PPARγ and RXRα, were also extremely potent inducers of adipogenesis (81, 82). Other studies have confirmed that receptor-specific activation of RXRα promotes both adipogenic differentiation and pre-adipocyte proliferation (60, 83, 84). Mechanistic experiments have further determined that of more than 5000 PPARγ:RXR DNA-binding sites in adipocytes, most are occupied by non-PPARγ:RXR heterodimers during the early stages of differentiation and transition to PPARγ:RXR in the later stages of differentiation (85). Mice with ablated adipocyte-RXRα are resistant to diet and chemical-induced obesity and exhibit impaired lipolysis during fasting (86); RXR agonists have also been demonstrated to sensitize diabetic and obese mice to insulin (87) and decrease hyperglycemia, hypertriglyceridemia, hyperinsulinemia, and both weight gain and food intake in several rodent models (87–89). More recent work from the Blumberg lab elegantly described RXR activation as an essential signal for commitment of mesenchymal stem cells to the adipocyte cell lineage, as well as separately promoting subsequent differentiation (48). Follow-up investigation determined that RXR activation-induced adipocyte differentiation created a functionally distinct adipocyte relative to those induced by PPARγ activation; RXR activation resulted in decreased glucose uptake, expression of adiponectin, and did not induce molecular pathways involved in adipocyte browning, suggesting a dysfunctional white adipose tissue that could potentially contribute to elevated obesity and/or diabetes risk (90). Therapeutic treatment by rexinoids in humans has reported increased plasma triglycerides, increased plasma cholesterol, and decreased thyroid hormones (91–93).

### Liver X Receptor (LXR), Constitutive Androstane Receptor (CAR), Pregnane X Receptor (PXR), and Farnesoid X Receptor (FXR)

LXR, CAR, FXR, AND PXR are permissive binding partners with RXR, forming receptor heterodimers that can be activated by ligands for either receptor or both (potentially resulting in a synergistic effect), reviewed in Shulman et al. (94). LXRα is expressed primarily in the adipose, liver, intestine, and kidney, while the β isoform is ubiquitously expressed; LXRs mediate cholesterol transport, stimulating cholesterol efflux from macrophages, promoting transport in serum and uptake into liver, increase degradation of cholesterol into bile acids, inhibit absorption in the intestine, and synthesize fatty acids and triglycerides (94). Some disparate results have been reported *in vitro:* Hummasti et al. reported that LXR agonists failed to promote triglyceride accumulation and/or adipocyte differentiation in 3T3-L1 cells and 3T3-F442A cells, though did regulate adipocyte-specific gene expression (95). However, other studies have described LXR-mediated promotion of triglyceride accumulation, adipocyte differentiation, adipocyte-specific gene expression, and pre-adipocyte proliferation both *in vitro* and *in vivo* (60, 96, 97), potentially via activation of PPARγ (96, 97). These disparate results could be explained by cells lines and/or cell sources, as we previously reported different LXR expression and responsiveness in varying pre-adipocyte sources (60). Selective knockdown experiments have demonstrated LXRα as the primary regulator of lipolysis (98), with the β isoform more involved in cholesterol regulation (99). LXRβ-specific knockout mice have less adipose, but normal insulin sensitivity and adipocyte hormones; however, they are glucose-intolerant and accumulate lipid in pancreatic islets, putatively mediated by regulation of cholesterol transporters (99). Adipocytes have been demonstrated to be smaller in LXR deficient mice (97), and energy expenditure is increased, with reduced triglyceride accumulation in brown adipose (100); in parallel, energy expenditure is reduced in LXR agonist-treated wild type mice, and triglyceride accumulation was increased in brown adipose (100). In humans, LXR expression is higher in obese individuals, and receptor isoform polymorphisms have been associated with increased risks of obesity (101). Therapeutic treatment with LXR agonists resulted in increased plasma and hepatic triglycerides, cholesterol, and other negative metabolic markers in humans as well as primate and rodent models (102), despite some beneficial effects.

CAR and PXR are two closely-related liver-enriched receptors that have also been associated with metabolic function, and were reviewed in detail previously (103, 104). While originally appreciated as regulating xenobiotic metabolizing enzymes, they have also been demonstrated to help regulate energy homeostasis, immune function, lipid metabolism, and glucose homeostasis (103, 104). PXR appears to mediate effects through PPARγ, with PXR activation directly inducing PPARγ and other lipogenic gene expression such as Cd36, though potentially in a species-specific manner (104). CAR may promote effects on energy homeostasis through crosstalk with PPARα, or similarly to PXR, through activation of the free fatty acid uptake transporter Cd36 and inhibition of sterol regulatory element-binding protein (SREBP) (105). In animals, PXR ablation inhibits diet-induced obesity, insulin resistance, and fatty liver disease in various rodent models, suggesting PXR antagonism as a putative anti-obesogenic and anti-diabetic pathway (104, 106). PXR agonist treatment in mice promotes hepatic triglyceride accumulation, and constitutively active PXR mice exhibit enlarged and fatty liver disease, reviewed in (107). Treatment with CAR agonists, in contrast, enhances insulin sensitivity, improves glucose and lipid metabolism, and reverses diet-induced obesity in rodents, reviewed in (103). In humans, the CAR agonist phenobarbital has been reported to decrease plasma glucose levels and improve insulin sensitivity in patients with diabetes (103, 108, 109), and though PXR is particularly promiscuous, activation of PXR by rifampicin, statins, and other pharmaceuticals have been reported to induce hyperglycemia in patients and increase the risk of developing diabetes (106). While activation of CAR is seemingly more therapeutically beneficial relative to PXR, it also carries with it side effects such as liver hyperplasia and carcinogenesis (103), among other effects.

Modulation of FXR has also been assessed as it relates to adipogenesis and a potential therapeutic target in treating metabolic syndrome, reviewed in (110). Endogenously activated by bile acids, FXR regulates bile acid synthesis, enterohepatic circulation, lipid metabolism, and thus indirectly regulates other bile acid associated receptors, discussed in Prawitt et al. (111). Researchers have described that FXR is expressed in adipocytes from adult mice and in differentiated 3T3-L1 cells, but not in the undifferentiated pre-adipocytes (112). Treatment with an FXR agonist increased adipocyte differentiation in 3T3-L1 cells, whereas treatment with an FXR antagonist reversed this (112); FXR agonist treatment also enhanced insulin signaling and insulin-stimulated glucose uptake (113). Pro-apoptotic and anti-adipogenic effects of guggelsterone (FXR antagonist) have also been reported by other researchers (114). Treatment with an FXR agonist in mice with diet-induced obesity worsened weight gain and glucose intolerance, seemingly mediated through reduction of the bile acid pool size and energy expenditure (115). However, other research in mouse models suggests beneficial effects for FXR agonist (GW4064) treatment (111, 116). FXR knockout/deficient mice exhibit decreased adipose tissue, lower leptin concentrations, elevated plasma free fatty acids, resistance to rosiglitazone-induced obesity, and their embryonic fibroblasts are also resistant to rosiglitazone-induced triglyceride accumulation and differentiation due to increased lipolysis and decreased lipogenesis (113, 117, 118); despite these apparent positive metabolic effects, FXR deficient animals (both mice and rabbits) also exhibit impaired glucose tolerance and insulin resistance, which are corrected with FXR agonist supplementation (113, 119). FXR expression has also been demonstrated to be downregulated and/or dysfunctional in obese humans (110, 120), suggesting downregulation may play a potential role in human obesity. PXR mice exhibit FXR agonist therapeutic trials in humans have reported reduced liver lipid accumulation and increased glucose uptake [reviewed in (110)], reduced HDL cholesterol and increased LDL cholesterol, and improvements in insulin resistance [reviewed in (111)], suggesting that FXR antagonists and/or selective FXR receptor modulators might promote more beneficial effects in some tissues and for specific metabolic endpoints (116).

### Thyroid Receptor (TR)

TR also forms a heterodimer with RXR, though in contrast to other receptors discussed above, it is considered a non-permissive heterodimer (can be activated only by thyroid receptor ligands and not RXR ligands), reviewed in Shulman et al. (94). While less frequently assessed as a contributory molecular pathway for adipogenesis, one of the defining characteristics of thyroid hormone action is maintenance of metabolic health and maintenance of lipid and carbohydrate metabolism, blood pressure, and body mass [reviewed in (94, 121)]. Hypothyroidism (low thyroxine (T4) and triiodothyronine (T3), high thyroid stimulating hormone (TSH)) is characterized by weight gain, while hyperthyroidism (high T4 and T3, low TSH) is characterized by weight loss (122, 123). As such, thyroid hormones are generally considered anti-obesogenic, and hypothyroid-associated adiposity can be reduced with supplementation (124–126). TRα primarily regulates thermogenesis and TRβ primarily regulates cholesterol metabolism and lipogenesis, as well as a number of genes and enzymes necessary for pre-adipocyte proliferation and adipocyte differentiation, either directly or via PPARγ [reviewed in (121)]. Studies have demonstrated some disparate findings regarding the role of TR in adipogenesis itself. For example, antagonism of TR has been demonstrated to efficaciously modulate adipocyte differentiation, purportedly via PPARγ, reviewed in (16); however, we've previously demonstrated that 3T3-L1 treatment with 1–850 (TR antagonist) resulted in efficacious triglyceride accumulation (60), and TR-null mice exhibit increased adipogenesis (127). Others, in contrast, have reported that treatment with triiodothyronine (T3; TR agonist) promoted adipocyte gene expression and decreased pre-adipocyte proliferation in Ob L771 mouse pre-adipocytes (128) or triglyceride accumulation and lipogenic gene expression in 3T3-L1 pre-adipocytes (60, 129). Other work suggested differing roles at varying levels of treatment; when 3T3-F442A cells were treated with hyperthyroid T3 levels, the proportion of adipocytes was increased but expression of lipogenic enzymes and triglyceride accumulation were decreased, whereas lower levels stimulated adipose conversion, expression of lipogenic enzymes, and pre-adipocyte proliferation (130).

### Glucocorticoid Receptor (GR)

The GR is intimately connected to lipid metabolism, with a wealth of *in vitro, in vivo*, and human epidemiological evidence supporting its role in adipose formation and maintenance [reviewed in (131)]. Treatment with dexamethasone induces a potent and efficacious triglyceride accumulation and pre-adipocyte proliferation response in various mesenchymal and pre-adipocyte models, often to greater extents and at lower concentrations than through direct activation of PPARγ (60, 132), potentially mediated at least in part through activation of PPARγ (133), though in other cases without meaningful activation of PPARγ (134); in support, treatment with GR antagonists inhibits differentiation in various mesenchymal and pre-adipocyte models (135). Other studies have reported that glucocorticoids alone were insufficient to promote adipogenesis either in 3T3-L1 cells (136) or in other models (137), though stimulated robust differentiation in combination with insulin (137). As mentioned above, Chappell et al. demonstrated putative GR-mediated effects prior to PPARγ activation after exposure to tetrabrominated bisphenol A (TBBPA) (61), which may explain why isobutylmethylxanthine (IBMX; PPARγ ligand) treatment prior to dexamethasone (GR agonist) failed to induce significant differentiation using the same cell model in another lab, while dexamethasone treatment before IBMX promoted robust differentiation (138). The authors posited that glucocorticoid activation may be necessary for an intermediate commitment state prior to differentiation via PPARγ (138); however, this could also be due to differing responsiveness to PPARγ and GR ligands based on 3T3-L1 cell source, which we have reported on previously (60).

Other research has evaluated the putative role of the mineralocorticoid receptor (MR), an additional high-affinity binder of glucocorticoids; treatment of 3T3-F442A and 3T3-L1 cells with the mineralocorticoid agonist aldosterone promoted adipocyte differentiation, which appeared to be mediated through PPARγ activation; inhibition and knock-down of the MR inhibited adipogenesis, whereas knock-down of the GR did not (139). More recent work, however, demonstrated that silencing GR, but not MR, inhibited the pro-adipogenic activity of cortisol, and also decreased leptin and adiponectin, whereas MR knock-down actually increased leptin (140). Research in mice investigated knocking out local glucocorticoid action via 11β-hydroxysteroid dehydrogenase (glucocorticoid inactivator) overexpression exhibited resistance to diet-induced obesity/reduced fat accumulation, decreased food intake, improved insulin sensitivity and glucose tolerance, and increased energy expenditure (141). Glucocorticoid excess in mice in contrast resulted in decreased osteogenic gene expression and mineralization and increased expression of adipogenic genes (142). Cushing's syndrome (excess cortisol production) is associated with increased weight gain, hypertension, type 2 diabetes, and fatty tissue deposits (143, 144), suggesting a pro-adipogenic effect of glucocorticoids in humans as well. Further, prenatal/antenatal dexamethasone (GR agonist) is often utilized to promote development of lungs in infants at risk of being born premature (145, 146). Epidemiological studies have reported that dexamethasone treatment is associated with reduced birth weight in infants, even after correcting for weeks of gestation (145, 146), and exhibited hypertension and greater subsequent administration of insulin for hyperglycemia (146).

### Estrogens and Androgens

Often considered opposing sex steroids, androgens, and estrogens have also been described to have opposing effects on adipogenesis, reviewed in Cooke and Naaz (147). Experiments comparing differentiation extent in rat pre-adipocytes determined no effects for either androgens or estrogens in promoting differentiation in male pre-adipocytes; however, estrogens elicited a pro-adipogenic effect (via pre-adipocyte proliferation) and androgens elicited an anti-androgenic effect in female cells, potentially mediated by modulation of insulin growth factor 1 receptor (IGF1R) and PPARγ expression (148). This promotion of pre-adipocyte proliferation by estrogens has been successfully replicated in both male and female omental pre-adipocytes (149), while the inhibitory effect of estrogens on differentiation/triglyceride accumulation may be dose-dependent (150). Related work has determined some of the inhibitory effects of estrogens on adipogenesis appear to occur through the G-protein-coupled estrogen receptor 1 (GPER) rather than the classical estrogen receptor itself (151), and that inhibitory effects on adipogenesis are concurrent with enhancement of osteogenesis (152). Interestingly, estrogen receptor knock-out (ERKO) mice exhibit increased fat pad weights, adipocyte size, and adipocyte numbers relative to wild type control animals, as well as insulin resistance and impaired glucose tolerance (153). This is mirrored in humans, as decreased estrogen levels at menopause are associated with increased abdominal obesity that is ameliorated with estrogen replacement therapy [reviewed in (154)], an effect also observed in ovariectomized female mice (155).

Androgens are generally considered anti-obesogenic [reviewed in (156, 157)], and treatment with androgens has been demonstrated to inhibit adipogenesis in adipose tissue samples from both sexes (158) and reduce fat mass in humans [reviewed in (159)]. Dihydrotestosterone inhibits triglyceride accumulation and adipocyte gene expression in human mesenchymal stem cells and pre-adipocytes from various depots, whereas anti-androgen co-treatment attenuated those effects, and had no apparent impact on pre-adipocyte proliferation in either model (160). Other research has replicated these findings, suggesting some of the effects occur through inhibition of the multipotent stem cell to pre-adipocyte commitment (161, 162). In contrast, anti-androgens have been suggested to act as obesogens; androgen receptor knock out (ARKO) mice exhibit increased obesity (163), flutamide has been demonstrated to modulate lipid profiles in women (164), and hypogonadism (characterized by testosterone deficiency) is associated with obesity, hypertension, dyslipidemia, insulin resistance, and other metabolic effects, which may be corrected with androgen supplementation (159).

### Other Receptors

A variety of other receptors, from the nuclear receptor family, receptor tyrosine kinase family, and others, have described roles in adipogenesis and/or lipogenesis. For example, both the insulin and IGF-1 receptors have widely accepted roles in growth, tissue-specific hypertrophy, and weight maintenance (165–168). Many others, including the aryl hydrocarbon, retinoic acid, low density lipoprotein receptors, among others, have established roles in adipogenesis but could not be discussed in detail within the scope of this review. Importantly, while the bulk of study has assessed activation of PPARγ and RXR, numerous other receptor systems interplay to promote and maintain adipocytes, and must be taken into account when evaluating environmental mixtures.

## Mitochondrial Toxicity as a Contributory Factor to Metabolic Disruption

Mitochondria are the major location of fatty acid oxidation, making them essential in lipid metabolism; as such, dysfunction can contribute to numerous adverse metabolic health consequences, including altered lipid accumulation, metabolism, and insulin resistance (169, 170). Mitochondrial function is intimately connected with metabolic health, as it helps regulate energy expenditure, production of ATP, and removal of reactive oxygen species (ROS); ROS reduce oxygen consumption and inhibit fatty acid oxidation in adipocytes, promoting lipid accumulation [reviewed in (169)]. ROS production mainly occurs at complex I and III in mitochondria, and is increased when excess electrons are provided to the mitochondrial respiratory chains (when proton gradient is high and ATP demand is low), as described in Kim et al. (171). Excess electrons are transferred to oxygen, converted to superoxide, and subsequently to hydrogen peroxide; this ROS acts to damage proteins, DNA, and lipids, and activates pathways (via activation of serine kinases) that phosphorylate insulin receptor substrate proteins and inhibit insulin signaling, thus promoting insulin resistance and ultimately resulting in metabolic dysfunction (171, 172). Mitochondrial dysfunction and resultant lipid accumulation in accessory tissues is also capable of further impeding insulin signaling and glucose metabolism, promoting further dysfunction (173); indeed, maternal obesity during pregnancy in rodents contributes to a transgenerational mitochondrial dysfunction phenotype (inhibited insulin signaling for three generations) (174). Notably, chronic oxidative stress has been well-described in obese individuals, suggesting a link between ROS production/management and hyperplasia [reviewed in (175)]. To minimize damage from these ROS, cells require a balance between ATP synthesis through oxidative phosphorylation and dissipation of the proton gradient (169). Mitochondrial dysfunction can also directly contribute to cardiovascular disease, another hallmark disease of metabolic syndrome, and myocardial metabolic function is intimately connected to obesity, diabetes, and altered insulin signaling [reviewed in (176)]. Research suggests that decreases in ATP production due to inhibited mitochondrial respiration, increased oxidative stress, and inhibited calcium signaling can all contribute to diastolic dysfunction via reduced velocity of myocardial relaxation velocity and myocardial compliance (173, 176).

Adipocytes are capable of regulating metabolic insults by altering their number, morphology, as well as the intracellular mitochondrial distribution (169). Mitochondrial biogenesis is an essential component of adipogenesis, with mitochondrial numbers increasing markedly after initiation of pre-adipocyte differentiation and reaching a maximum toward the end; this can be noted via treatment with the PPARγ agonist rosiglitazone, wherein treated cells demonstrate increased mitochondrial content and function, with increased basal oxygen consumption, ATP respiration, and proton leak (173, 177, 178). ATP levels are naturally reduced with increasing degree of adipocyte differentiation, putatively due to increased ATP demands for lipogenesis (179), and reduced levels are further exacerbated when electron transport chain inhibition occurs (178). Reduced mitochondrial biogenesis, ATP levels, and dysfunctional mitochondrial electron transport have been reported in both humans and animals with metabolic syndrome (173, 179). PPARγ co-activator 1α (PGC-1α) is a master regulator of mitochondrial biogenesis and gene expression and is a potent co-activator of PPAR isoforms: expression in fat or muscle cells increases mtDNA content, expression of mitochondrial genes, and mitochondrial respiration (176). PGC-1α-stimulated biogenesis in the heart ultimately promotes overt heart failure, another mechanism through which metabolic dysfunction can lead to cardiac dysfunction (176). Biogenesis appears closely linked to adipocyte differentiation, as up-regulation of mitochondrial biogenesis is well-reported following induction of adipogenesis and for up to 10 days post-differentiation (177, 180), suggesting that mitochondria are needed to supply the substrates and factors necessary to support adipogenesis-driven lipogenesis. Mitochondria also have functionally distinct roles in white vs. brown adipose tissue. White adipose tissue is composed of numerous depots of large lipid droplet adipocytes throughout the body and is essential for maintenance of metabolic health. Brown adipose tissue, in contrast, is smaller and localized to the neck and upper-chest in adult humans, and is composed of adipocytes with large numbers of smaller lipid droplets and more numerous mitochondria [reviewed in (180)]. In brown adipose tissue, the heat derived from thermogenesis is produced primarily by the high mitochondrial content of these cells, via oxidation of fatty acids and other components (180). Uncoupling proteins (UCPs) play a key role in this process, serving to uncouple mitochondrial respiration from ATP generation by inducing a proton leak, which subsequently allows for energy dissipation as heat (180).

Numerous environmental toxicants have been demonstrated to promote mitochondrial dysfunction, and these contaminants may also advance metabolic dysfunction, leading to obesity, and diabetes. Certain mitochondrial disorders that are characterized by impaired oxidative phosphorylation are also associated with disrupted lipid homeostasis: myoclonic epilepsy with ragged red fibers is associated with triglyceride accumulation in muscles and multiple symmetrical lipomatosis, a condition characterized by abnormally small white adipocytes containing numerous small lipid droplets rather than the classical large central droplet that displaces the nucleus (170). Mitochondrial oxidative phosphorylation inhibitors and protein synthesis inhibitors impair mitochondrial respiration and promote triglyceride accumulation in 3T3-L1 cells, which retain their precursor fibroblastic morphology and do not express adipocyte-specific markers (170, 180). Previous research has demonstrated that treatment of 3T3-L1 cells with rotenone (a complex I inhibitor), antimycin A, stigmatellin, and myxothiazol (complex III inhibitors), and oligomycin (ATP synthase inhibitor) promoted triglyceride accumulation in a dose-dependent manner (170). Interestingly, these mitochondrial respiration inhibitors promoted triglyceride accumulation in numerous small lipid droplets, cells retained their fibroblastic morphology, and classical adipocyte-specific genes were not expressed in these cells (170), suggesting a differentiation-independent mechanism of triglyceride accumulation. Specifically, antimycin A, which inhibits complex III, induces triglyceride accumulation in pre-adipocytes via a putative differentiation-independent mechanism (170); these cells exhibit multi-vesicular lipid accumulation, reduced expression of standard differentiation markers (FABP4, C/EBP), and suppression of PPARγ and RXR, supporting other studies suggesting mitochondrial dysfunction may inhibit adipocyte differentiation (178).

We recently demonstrated a similar phenotype in experiments with pyraclostrobin, a strobilurin-class fungicide used on strawberries, spinach, and other produce items, with production of >2 million pounds per year (181, 182). Pyraclostrobin and other strobilurin fungicides have been demonstrated to inhibit complex III (183), suggesting a potential mechanism for metabolic disruption. We previously reported that pyraclostrobin, azoxystrobin, fluoxastrobin, and trifloxystrobin all induced both triglyceride accumulation and pre-adipocyte proliferation in 3T3-L1 cells (184). Previous research in 3T3-L1 cells and a human adipose-derived stem cell model suggested this did not occur through activation of PPARγ and that standard differentiation markers were lacking (47, 185), supporting the case for a differentiation-independent mechanism. Mechanism was further interrogated in our laboratory through co-exposure experiments in 3T3-L1 cells; we reported that PPARγ antagonists did not protect against pyraclostrobin-mediated triglyceride accumulation (177). Instead, pyraclostrobin promoted mitochondrial dysfunction, including reduced ATP, mitochondrial membrane potential, basal mitochondrial respiration, ATP-linked respiration, and spare respiratory capacity (177). In addition, pyraclostrobin-treated cells exhibited reduced expression of genes regulating glucose transport, glycolysis, fatty acid oxidation, and lipogenesis (177). Lastly, co-treatment with a cAMP responsive element binding protein (CREB) inhibitor reduced pyraclostrobin-mediated triglyceride accumulation (177). These results all suggest that toxicants capable of disrupting mitochondrial function may also have the potential to affect metabolic health, via modulation of lipogenesis and other metabolic processes.

Similarly, a recent study reported that several samples of oil sands process-affected water (OSPW), wastewater produced during the extraction of bitumen from oil sands, exhibited PPARγ agonist activity and promoted triglyceride accumulation in 3T3-L1 cells (186). Causative ligand characterization identified several hydroxylated/polyoxygenated carboxylic acids and hydroxylated sulfates as the major PPARγ ligands (186); naphthenic acids, a mixture of carboxylic acids and natural component of petroleum, are a major component of OSPW. Interestingly, while these are posited as promoting adipogenesis via PPARγ activation, a recent publication demonstrated that naphthenic acids isolated from oil sands water acted to uncouple oxidative phosphorylation, inhibit respiration, and increase the production of ROS (187). As noted above, these are mechanisms that can promote triglyceride accumulation in cells, suggesting that this may be an additional mechanism for the observed adipogenic effects of these waters in the previous publication (186). Notably, this occurred at environmentally-relevant concentrations for OSPW, suggesting that this or a combination of these mechanisms may promote the environmental sample-induced adipogenicity.

Mitochondrial ROS is produced by pre-adipocytes during and throughout differentiation, and its presence activates several early-stage differentiation markers, including C/EBP, PPAR, and CREB (63, 188). Direct impacts on adipogenesis appear less certain, which has been delineated in greater detail previously (189); research suggests that ROS may be essential for adipogenesis, but also may perturb the process. Some research has demonstrated that ROS promoted mitotic clonal expansion in 3T3-L1 cells (190), a necessary step prior to induction of differentiation. Other research has described inhibitory effects on differentiation, with ROS inhibiting both pre-adipocyte proliferation and adipocyte differentiation/triglyceride accumulation (180, 191, 192). Still other researchers, via co-treatment experiments utilizing antioxidants, demonstrated that ROS impacted differentiation but not pre-adipocyte proliferation: treatment with an antioxidant (reducing ROS) reduced lipid accumulation in mesenchymal stem cells (193). However, the varying cell models used for these experiments may mediate these apparent differences; all studies agree that ROS appear to modulate early-stage differentiation, though the mechanisms of this modulation appear to vary based on cell lines, sources, and experimental details.

Several mitochondrial toxicants have been demonstrated to promote insulin resistance and/or metabolic syndrome in epidemiological studies, reviewed in detail previously (172, 194), though this research area has as of yet received limited attention in the context of metabolic disruption. Several organochlorine pesticides have been implicated in metabolic effects via mitochondrial dysfunction. Specifically, atrazine has been demonstrated to directly inhibit complexes I and III, reducing oxygen consumption and leading to accumulation of superoxides; chronic exposure in rats has been demonstrated to decrease basal metabolic rate, and increase body weight, intra-abdominal fat, and promote insulin resistance independent of food intake or activity levels (172, 195). Much of the remaining literature has focused on the role of polychlorinated biphenyls (PCBs). Several congeners have been demonstrated to promote mitochondrial dysfunction *in vitro* (196, 197), exposure resulted in/exacerbated obesity, insulin resistance, and hyperinsulinemia in mice (198), and higher exposure to PCBs has been linked to increased risk of obesity, dyslipidemia, and/or insulin resistance in a number of epidemiological studies (199–202).

## Available *in vitro* Models of Adipogenesis and Metabolic Disruption

Numerous *in vitro* models have been developed and utilized for the purpose of identifying potential metabolic disrupting chemicals, reviewed in detail previously (203, 204). Generally, these models can be described as assessing two key parameters of adipocyte development: commitment to the adipocyte lineage from multipotent precursor cells (generally through the use of mesenchymal stem cell (MSC) models) and differentiation into mature adipocytes (generally through the use of pre-adipocyte models). MSC models have the additional benefit of being capable of assessing both endpoints, though are seemingly less frequently utilized than the available pre-adipocyte models. In addition, several research groups have begun to report on the three-dimensional culture of pre-adipocytes, which may shed additional light on mechanisms in a more physiologically relevant system. All of these assays are lengthy and their relative abilities to correctly identify chemicals may depend on both cell line and cell source. As such, there is a critical need to develop better methods for correctly predicting metabolic disruptors. While murine models have historically been used preferentially, a growing number of species utilized and a growing movement toward utilization of human models may help expand our understanding of translational mechanisms and potential environmental contaminant impacts on human health.

Perhaps the best known pre-adipocyte model is the 3T3-L1 mouse cell line. First described in the 1970's, it has proven reliable as an *in vitro* screen over several decades for identifying likely obesogenic chemicals *in vivo* (205, 206). These cells are already committed to the adipocyte lineage and cannot develop into other cell types; however, they generally require activation of particular signaling pathways to promote further development. Following exposure to adipogenic chemicals, these cells differentiate into adipocytes, accumulate triglycerides, and come to resemble a mature human white fat cell (44, 46, 205, 206). While 3T3-L1 cells have seemingly come to be considered the de facto model of adipogenesis, some inherent concerns remain about their utility. As we have described recently (60), while this line has been well-characterized (207), it is somewhat unreliable in sourcing. For example, while we know much about the molecular mechanisms underpinning the development of mature adipocytes based on this cell line, nuclear receptor expression related to adipogenesis is markedly different between different lots and sources of this cell line (60). Moreover, on investigation into this apparent discord in source, we discovered that the American Type Culture Collection (ATCC) maintains five distinct lots of 3T3-L1 cells, which all seemingly have differing degrees of differentiation success. This issue with cell line integrity was highlighted in a recent paper (208), suggesting that these differences can contribute to real discrepancies in the ability to replicate findings across laboratories. As the current ATCC cells are meaningfully different in the expression of key adipogenic pathways from the Zenbio-sourced cells (which are sourced from the isolating laboratory), it is unclear whether our understanding of the mechanisms underlying adipogenesis are from the original cells, the ATCC cells, or where these research paths diverge. Care needs to be taken to assess reproducibility across stocks and between laboratories and carefully untangle where the research underlying this cell line belongs. Other pre-adipocyte models also exist, including the OP9 mouse bone marrow-derived stromal pre-adipocyte cell line (44, 45), a line that allows for considerably faster differentiation, though which we have demonstrated to exhibit different nuclear receptor expression and differing degrees of responsiveness to adipogenic chemicals (60). These varying pre-adipocyte models allow for assessments of varying molecular pathways important for the process of differentiation via both source of the cells and species [discussed further in (204)].

Various multipotent mesenchymal cells and cell lines (46, 47) offer the additional ability to assess commitment to the adipocyte lineage as a distinct process from adipocyte differentiation (48). MSC use and applicability in adipogenesis research has been reviewed in detail previously (209). The variability of these cell lines are reportedly lower than the pre-adipocyte models, they are purportedly easier to isolate and culture, and they have additional utility in that they can be utilized to assess both differentiation of adipocytes but also commitment to the adipocyte lineage vs. other cell lineages. For example, many researchers have utilized these cell lines to evaluate the interplay between commitment to the osteogenic vs. adipogenic lineages following exposure to specific environmental contaminants (210–213). Recent work elegantly described a novel protocol for evaluating both adipogenic lineage commitment and subsequent differentiation as distinct processes in primary MSCs (48), which has been described previously for the C3H10T1/2 stem cell model (214, 215). These advancements raise the utility of this model and warrants further investigation into replicability, reproducibility of this model across laboratories, and comparisons of translation to human health relative to the pre-adipocyte models currently utilized.

Lastly, a number of research labs have begun to describe spheroid cell cultures of adipocyte models (216–220), which may carry some inherent benefits over the standard adherent monolayer cultures. These studies have suggested that spheroid culture improves the efficiency, extent, and/or speed of differentiation (216–221), retains the multipotent potential of these cells (217, 222), and transcriptomic analyses have suggested a potentially more representative model of adipocyte gene expression relative to known *in vivo* mechanisms (216). These models may allow for a more comprehensive understanding of adipose physiology than was possible via interrogation of the monolayer cell cultures, and should be evaluated further for replicability and translation potential relative to the standard monolayer cultures.

## Metabolic Disruption Potential of Environmental Mixtures

**Figure 3:**
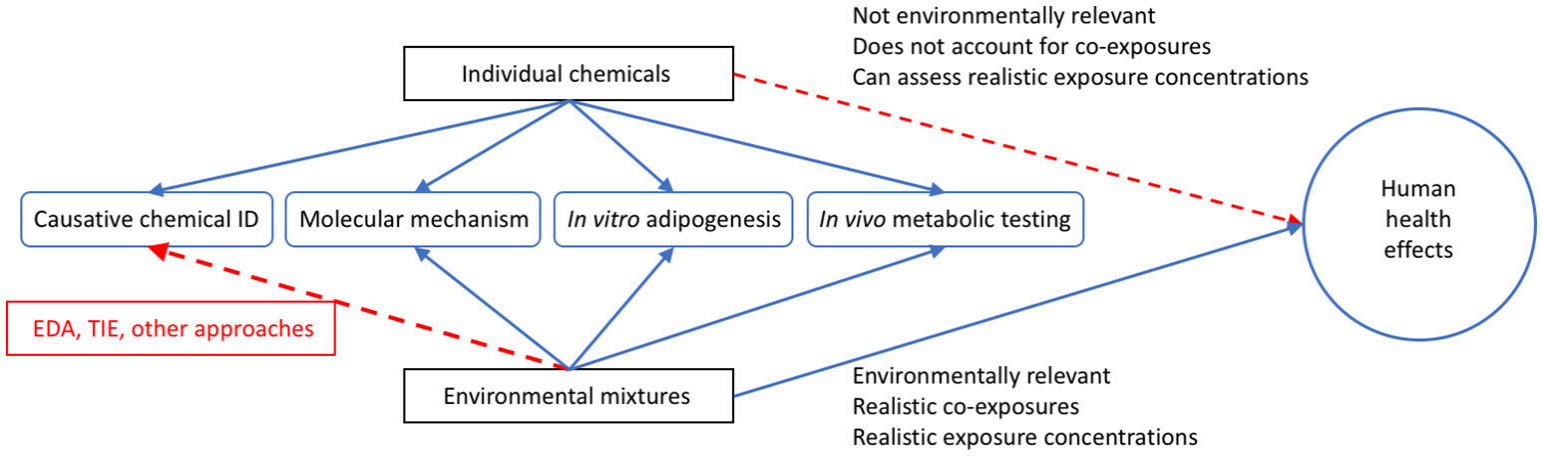
Utility of Utilizing Environmental Mixtures for Human Health Assessments. Graphical depiction comparing individual chemicals and environmental mixtures for the assessment of potential human health effects. While environmental mixtures have less use in identifying causative chemicals in all cases (though tools like effect directed analysis (EDA) and toxicity identification evaluation (TIE) can be used to elucidate this), these mixtures are more relevant in reflecting the suite of chemicals that people are exposed to on a daily basis than utilizing single chemicals alone, and more often reflect actual environmental exposure concentrations. In this figure, the blue lines indicate positive relationships and the red lines indicate difficulty for single chemicals or mixtures in assessing the related outcome.

As noted above, the assessments of environmental samples have proven an interesting new approach to evaluating potential mixture toxicity. With tens of thousands of chemicals in use and new chemicals regularly added, there are too many to characterize individually, and certainly no capabilities to assess all potential combinations of them (223, 224). Body burden studies have and continue to report human exposure to hundreds of chemicals on a regular basis (225, 226), demonstrating the problem of realistic mixture exposure studies. To add to the complexity, research has reported additive effects on several hormone receptors both *in vitro* and *in vivo* (227–231), demonstrating that mixtures can induce effects at levels below those induced by individual chemicals. From a toxicological perspective, evaluating whole environmental samples: wastewater, surface/groundwater, indoor house dust, air samples, etc. for biological activities has emerged as a promising tact to assess potential adverse health concerns from exposure to actual mixtures present in the environment, given that it can evaluate more realistic environmentally relevant exposures (Figure 3).

Numerous natural and exogenous contaminants can contribute to human exposure; as such, measuring the total receptor bioactivities has proven useful for assessing the total magnitude of potential effects (232–239). While analytical chemistry techniques and equipment have drastically improved, allowing for more precise measurements of contaminants at lower concentrations, recent research has suggested we lack complete information on all causative bioactive chemicals present in the environment (240, 241). While non-targeted analytical efforts have improved, we still lack sufficient software and comprehensive protocols to enable robust and reproducible non-targeted assessments of contaminants across laboratories. To address this need of addressing mixture toxicity without necessarily understanding the full chemical complexity, bioassays have been utilized to assess biological activities of actual environmental samples. Reporter gene assays are one such commonly-utilized tool, assessing total receptor activities (agonism and antagonism), and valued due to their low cost, ease of use, reliability, high sensitivity, and ease of adapting for multiple receptors (227–244). These assays provide the capability to assess the total receptor activity of potentially numerous low-concentration EDCs (without identifying each causative chemical) rather than assessing each constituent chemical individually.

Applying this method to human epidemiological research has shown great potential; a number of researchers have rigorously characterized how *in vivo* mixtures of contaminants correspond with total hormone receptor bioactivities of human and animal matrices (serum, tissues, etc.) (245–251). Moreover, some researchers have begun to utilize bioactivities directly to assess human health outcomes. For example, researchers have correlated the total placental estrogenic activity with increased reproductive malformations (252) and impaired motor development (253), total adipose estrogenic activity with increased risk for breast cancer (254), and placental estrogenic activity with increased birth weight in boys (255). Other research has failed to report significant associations, including a lack of any association between adipose estrogenic activity and risk for type-2 diabetes (256), potentially due to a greater role for other receptors in pathogenesis (257). These studies demonstrate the potential utility of this method, particularly when targeted based on a comprehensive understanding of etiology and molecular mechanisms.

Several studies have begun to apply these techniques to metabolic endpoints, assessing pertinent receptor bioactivities (GR, PPARγ, and others) as well as utilizing less high-throughput adipogenesis or other assays for predicting *in vivo* metabolic disruption potentials. Some of these environmental case studies are discussed in greater detail below:

### Metabolic Disruption Potential of Indoor House Dust

As noted above, numerous studies have documented the detection of EDCs from diverse chemical classes in indoor house dust samples from a variety of sources. A number of studies have assessed the bioactivities for solvent-extracted house dust, reporting PPARγ, GR, and ER agonist activities as well as AR and TR antagonist activities, at concentrations ≥ 15 μg dust equivalence per mL (DEQ/mL, mass of extracted dust per volume of assay medium) (258–260). Our laboratory also assessed the modulation of PPARγ by house dust extracts, reporting that 21 of 24 examined indoor house dust extracts exhibited significant PPARγ binding at 3 mg DEQ/mL (120 μg dust per assay well) using a relative binding affinity assay (261) and 15 of 25 extracts activated PPARγ at ≤ 50% of the maximal positive control response at concentrations ≥100 μg DEQ/mL (4 μg/well) using a commercially-available reporter assay (262, 263). This work demonstrated activation of pathways known to regulate adipogenesis at very low concentrations, and subsequently informed our follow-up studies examining higher-order effects on adipogenesis.

We recently evaluated >40 common SVOCs that are routinely detected in indoor house dust samples for adipogenic activity in the 3T3-L1 murine pre-adipocyte cell model. We found that > two-thirds of these chemicals independently induced significant triglyceride accumulation and/or pre-adipocyte proliferation (184). Specifically, pyraclostrobin (strobilurin fungicide), dibutyl phthalate (DBP), tert-butyl-phenyl diphenyl phosphate (TBPDP), and the isopropylated triaryl phosphates (ITPs, mixture of isomers) exhibited near or supra-maximal triglyceride accumulation relative to the rosiglitazone (positive control)-induced maximum (184). We further assessed eleven house dust extracts collected from central North Carolina (NC), USA households; we found that ten of these 11 extracts exhibited significant triglyceride accumulation and/or pre-adipocyte proliferation at < 20 μg of dust/well (184). This activity occurred at orders of magnitude lower concentrations than those the EPA estimates children to consume each day. As such, this raises concerns for potential impacts on *in vivo* metabolic health.

A recent follow-up to this study evaluated the adipogenic activity of 137 house dust extracts from central NC households and attempted to determine putative causative chemicals, molecular mechanisms, and potential impacts on human metabolic health (264). We reported that 90% of the dust extracts exhibited significant adipogenic activity, < 60% via significant triglyceride accumulation, and >70% of samples via significant pre-adipocyte proliferation, with >40% of effects occurring at < 10 μg dust/well (264). Increasing dust-induced triglyceride accumulation was positively correlated with serum thyroid stimulating hormone levels in adult residents, and negatively correlated with serum free triiodothyronine (T3) and thyroxine (T4) (264). Interestingly, proliferation tended to be positively correlated with residents' body mass index (BMI; *p* < 0.10), potentially suggesting adipogenic chemicals present in the dust are associated with the weights of residents, but further research with larger sample sizes are needed to substantiate this. We further assessed TR antagonism as a potential contributory causative mechanism in these effects, and found that TRβ antagonism of these extracts (265) was positively correlated with triglyceride accumulation (264). Both T3 co-treatment and siRNA knock-down of TR inhibited the dust-induced triglyceride accumulation of these extracts, supporting the role of TR antagonism as a contributory molecular mechanism.

### Metabolic Disruption Potential of Oil and Gas-Associated Wastewaters

Three separate sets of studies have assessed different aspects of oil and gas operations and metabolic disruption, reporting *in vitro* and/or *in vivo* evidence of metabolic disruption by oil and gas associated environmental mixtures. The first assessed three replicate samples of oil sands process-affected water (OSPW), wastewater produced during the extraction of bitumen from oil sands (186). They reported that an OSPW sample activated PPARγ at concentrations as low as 0.025x relative water concentration (40-fold dilution relative to pure water). This sample was further fractionated, with the majority of PPARγ activity in fractions two and five (five fractions), and fractions three through five exhibited significant triglyceride accumulation and induction of adipogenic genes (fatty acid binding protein and lipoprotein lipase). A pull-down assay and chemical analysis was further utilized to identify the causative ligands present in fraction five that were inducing the adipogenic effects; this analysis revealed hydroxylated/polyoxygenated carboxylic acids and hydroxylated sulfates as the major PPARγ ligands inducing adipogenesis in these samples (186), though the small sample size requires further substantiation.

Another set of studies assessed the metabolic disruption potential of crude oil singly or mixed with Corexit oil dispersant mixture (266, 267). To distinguish these mixtures, they utilized several simpler mixtures in culture media, including: Corexit 9500 + MC252 oil, varying dilutions of MC252 oil, and varying dilutions of Corexit with corn oil; they found that the Corexit + oil treatments stimulated PPARγ, while the MC252 oil alone did not, suggesting a component of Corexit promoting the observed effects (267). The Corexit + oil mixture was further fractionated to determine causative ligands, with Tween 80 and dioctyl sodium sulfosuccinate (DOSS) identified as highly abundant chemicals in the active fraction (267). DOSS was further demonstrated to be active in PPAR response element-luciferase transgenic mice and stimulate triglyceride accumulation and expression of fatty acid binding protein (Fabp4) in 3T3-L1 cells (267). Follow-up work assessed the Corexit + oil mixture and Corexit alone for activation of RXRα, finding dose-dependent activation, presumably mediated by Corexit constituents (266). Constituent chemicals were further evaluated, and DOSS, Span 80, and Tween 80 all demonstrated some degree of RXRα activity, with Span 80 also stimulating triglyceride accumulation and adipocyte gene expression in 3T3-L1 cells. Interestingly, a combination of DOSS and Span 80 resulted in putative synergistic effects on adipocyte differentiation, potentially due to diverging molecular mechanisms (Span 80 exhibited a much more efficacious response for RXRα than PPARγ, while DOSS exhibited no RXRα activity but did activate PPARγ) (266).

The last set of studies, from our laboratory, evaluated unconventional oil and gas associated wastewater and chemicals. Our work on this topic began with receptor activity testing for 24 common hydraulic fracturing chemicals, reporting that 21 and 7 chemicals antagonized AR and TR in two cell-based assays, and that mixtures of these chemicals appeared to act synergistically for TR and additively for AR (36, 268). We further documented AR and TR antagonist activities in surface, ground, and/or drinking water near UOG operations in several regions, including CO, WY, WV, and ND [(268), Kassotis et al., in preparation, (269–271)], and evaluated a mixture of 23 common UOG chemicals via a gestational exposure experiment in C57 mice, reported putative metabolic effects (offspring exhibited increased body weights, among other effects) (36, 37). We further interrogated this by evaluating the ability of this 23-mix, several UOG wastewater samples, and several UOG wastewater-impacted surface water samples to stimulate adipogenesis in 3T3-L1 cells and activate PPARγ in a reporter gene assay (35). We demonstrated that UOG wastewater samples exhibited significant triglyceride accumulation and/or pre-adipocyte proliferation at relative water concentrations as low as 0.001x, UOG-impacted surface water extracts at concentrations as low as 0.04x, and the 23-mix at 1 μM; these effects co-occurred with PPARγ activation for some samples but not others (35), suggesting differing mechanisms. Related work demonstrated highly efficacious triglyceride accumulation for various non-ionic alkylphenol and alcohol polyethoxylates in the absence of PPARγ activation and potentially mediated by TR antagonism (272). These compounds are reportedly found at high concentrations in UOG wastewater (273–275) and may be responsible for some of the observed non-PPARγ-mediated effects observed in the UOG samples.

## Potential Utility of High-Throughput Databases to Predicting Metabolic Disruption

The costs and time investments associated with *in vivo* examination of putative metabolism disruptors are prohibitively high; as such, utilizing lower-order testing and screening is critical to target higher-order testing on chemicals most likely to be active. Application of numerous *in vitro* models for assessing putative “obesogens” or “metabolic disruptors” over the last several decades has revealed numerous contaminants capable of affecting metabolic health (18), with recent publications suggesting that these contaminants are likely common in indoor and outdoor environments (2, 184, 272). While these pre-adipocyte and mesenchymal stem cell models are useful in determining potential *in vivo* metabolic disruptors, they are also time and energy intensive and their relative abilities to correctly identify chemicals may depend on both cell line and source. Further, their mechanisms of assessing adipogenic commitment, adipocyte differentiation, adipocyte proliferation, and/or lipid accumulation may not capture the full spectrum of endpoints that compose metabolic dysfunction more broadly, particularly endpoints related to “diabetogens”. As such, there is a critical need to develop better methods for correctly predicting metabolic disruptors, and while more simplistic models such as activation of PPARγ are often applied, the vast suite of mechanisms influencing this process (discussed above) require a more holistic approach to integrating causative molecular mechanisms. Several high-throughput (HTP) screening programs now exist (Tox21, ToxCast) that report activity across numerous molecular mechanisms for thousands of chemicals, many that are known to be relevant to metabolic health. Harnessing these data sets to broadly assess high-scoring chemicals (across relevant molecular pathways for select endpoints of interest) for more targeted higher-order testing may provide a valuable tool for reducing time and research costs and achieving a more broad assessment of the tens of thousands of commercial chemicals for potential contribution to adverse health outcomes in humans and/or animals.

This issue of utilizing HTP data in predictive models is not new and has been applied by a number of researchers to various *in vivo* endpoints, with varying degrees of success (276). Most of these methods have utilized ToxCast Phase I data, due to the more recent release (October 2015) of Phase II results, and as a result, some of the inherent issues reported by these studies have since been addressed. For example, Schwarzman et al. attempted to build a model to predict breast carcinogens, though had insufficient data on particular endpoints critical to altered mammary development (277). Many of the pathways missing, including prolactin, progesterone, and estrogen receptor beta effects, among others, are now pathways with associated assays in the Phase II database. Russell et al. applied a broad approach to predicting 60 *in vivo* endpoints, 56 of which were predicted at < 55% accuracy (278), though notably did not aggregate assays to predict *in vivo* endpoints. Given that health outcomes are nearly always driven by overlapping molecular pathways, this is not altogether surprising. Other researchers utilized assay aggregation and were more successful in building predictive models that performed with promising accuracy (>70%). Martin et al. utilized a suite of ToxCast assays to develop a predictive model for rat reproductive toxicity, achieving ~75% accuracies for training and test sets (279). Notably, this model incorrectly predicted five of 21 external validation chemicals as predicted negatives, all of which reduced early offspring survival with limited accompanying effects on reproductive performance or reproductive tract development, suggesting a gap in assays targeting these endpoints. Another model applied ToxCast data to rat prenatal developmental toxicity, with >70% accuracy with species-specific models (280), and found that if they further refined this to more specific developmental outcomes, they got even better predictive success (80–90%). Liu et al. utilized both Phase I and Phase II data to predict hepatotoxicity (hypertrophy, injury, and proliferative lesions), and reported 53–61% accuracy using only Phase I data, but >80% when utilizing the expanded Phase II data (281).

Recently, Auerbach et al. presented predictive models of putative obesogenic and/or diabetogenic chemicals through analyzing ToxCast HTP results (282). The researchers, utilizing experts in a diversity of metabolic health disciplines, selected known molecular pathways that had been previously demonstrated to modulate metabolic health, and combined them into a combined score metric for predicting likely vs. less-likely metabolic disrupting chemicals. Janesick et al. recently tested a portion of this method, utilizing a suite of assays deemed relevant for adipocyte differentiation (16 assays across 8 molecular mechanisms) to assess 24 chemicals (11 with highest activation scores across the selected assays, 6 with medium activation scores, and 7 presumed negative controls with low activation scores) for activation of RXRα, PPARγ, and triglyceride accumulation in 3T3-L1 cells (47). They reported that 7 of 17 high and medium-scoring and 2 of 7 low-scoring chemicals were active in 3T3-L1 cells, suggesting poor predictivity (high rates of both false positives and false negatives). The authors suggested several potential hypotheses for the poor performance, including: poor performance of PPARγ assays, incorrect selection of assays for the predictive model, and improper weighting of endpoints (rather than based on mechanism importance) and assays within each endpoint (rather than based on assay performance).

We recently undertook an effort to improve the predictive utility of this model by expanding the pathways and attempting to incorporate some of the suggestions made by Janesick et al. (47). Among these, we expanded the outcome by performing a targeted literature search on all chemicals and any evidence of effects on metabolic health. This model performed best when used as a gross metabolic disruption prediction model, using literature searches to identify any *in vitro* or *in vivo* evidence of adipogenesis or disrupted metabolic health (weight gain, adipose development, insulin/glucose signaling, effects on appetite/satiety, etc.). When applied to a novel set of chemicals for which we had assessed adipogenic activities in 3T3-L1 cells (60, 184), the original prediction model performed well at predicting gross metabolic disruption; we observed low rates of both false negatives (7.9%) and false positives (7.9%), and an apparent accuracy of 84% (283).

We also attempted to bolster this model through inclusion of additional pathways known to modulate metabolic health, in hopes of reducing false negatives, though discovered that expanding the model to incorporate all of these pathways would produce an inappropriately large and unwieldy model with a considerably inflated false positive detection rate. Nonetheless, we determined that additional pathways could be incorporated into the model if there were a better method for de-selecting less important or artifactual pathways. Z score corrections were designed to address this by removing the bioactivities nearest cytotoxicity as presumed false negatives/non-specific effects. In our analysis, utilizing the cytotoxicity-derived z score values to remove putative cytotoxicity-impacted pathways was effective at reducing false positives, but at the expense of increasing false negatives. We determined that utilizing Z score corrections (even with a low threshold) was not an effective option to clarify important pathways and reduce false positives.

Results from these publications suggest that further improvements should focus on bolstering molecular pathways with poor-performing assays or where replicate experiments and/or assays are not available for a given endpoint within ToxCast. Ensuring data integrity and robustness is of profound importance to correct predictions. Efforts such as this have tremendous putative utility, as screening all chemicals and mixtures of chemicals for all endpoints is not feasible, and determining a screen of HTP assays could save tremendous time and cost and allow for a dramatically narrowed scope of testing *in vivo*. Further testing is required to substantiate this adipogenic prediction model for predicting *in vivo* metabolic disruption across a larger chemical space, but these preliminary results and success with other complex biological effects demonstrate a clear potential for implementation into predicting metabolic disruption and potentially helping reduce and better target *in vitro* and *in vivo* chemical assessments in the future.

## Author Contributions

CK and HS planned and outlined the proposed review. CK wrote the review, and HS read and bolstered the review via feedback and guidance.

### Conflict of Interest Statement

The authors declare that the research was conducted in the absence of any commercial or financial relationships that could be construed as a potential conflict of interest.
